# Frequency of typhoid-related intestinal perforation with atypical clinical presentation at Tertiary Care Hospital in Pakistan

**DOI:** 10.12669/pjms.41.12.12031

**Published:** 2025-12

**Authors:** Tahniyat K. Rizvi, S. M. Ashraf Jahangeer, Aasia Yousuf, Ghansham Rawtani

**Affiliations:** 1 Tahniyat K. Rizvi, Postgraduate resident General Surgery Department, Jinnah Postgraduate Medical Centre (JPMC), Karachi, Pakistan; 2 S. M. Ashraf Jahangeer, Assistant Professor, Department of Community Medicine, Dow University of Health Sciences, Karachi, Pakistan; 3 Aasia Yousuf, Consultant General Surgeon, Jinnah Postgraduate Medical Centre (JPMC), Karachi, Pakistan; 4 Ghansham Rawtani, Assistant Professor of General Surgery Department, Jinnah Postgraduate Medical Centre (JPMC), Karachi, Pakistan

**Keywords:** Atypical clinical presentation, Typhoid fever, Typhoid associated intestinal perforation

## Abstract

**Objective:**

To determine the frequency of cases with atypical clinical history diagnosed as typhoid-related intestinal perforation (TIP) in a tertiary care hospital.

**Methodology:**

A Cross sectional study was conducted in department of General Surgery, Jinnah Postgraduate Medical Centre, Karachi, from 20^th^ January 2021 to 20^th^ January 2024. Ninety-eight patients meeting the inclusion criteria of age 13-75 and diagnosis of typhoid related intestinal perforation, were enrolled through non-probability consecutive sampling. Data was collected after taking an informed verbal consent in self-designed proforma. SPSS version 25 was utilized for data analysis. A P-value of ≤ 0.05 was considered as significant.

**Results:**

Median age was 23 (18, 35) years with 82.7 % male predominance. Out of ninety-eight cases, 96.94% (95) had at least one atypical clinical feature while 23.49% (23) had all three atypical features at presentation. About 75.5% (74) of the cases had atypical duration of illness (≤14 days or >21 days). 76.53% (70) had atypical fever pattern (other than continuous step ladder). 5.1% (5) were afebrile, also considered as atypical. 41.84% (41) had atypical bowel habits (normal). Most common time of presentation was week-one 38.8% (38), followed by week-three, week-two and beyond week-three. The most common fever pattern was intermittent 66.3%, followed by continuous, remittent and relapsing. Constipation was more common 58.16% (57) than diarrhea 41.84% (41).

**Conclusion:**

Majority of patients with TIP had short duration of illness and intermittent pattern of fever, which were prone to be misdiagnosed due to their atypical presentation. Such cases must come into consideration for timely and adequate management of the disease.

## INTRODUCTION

Enteric fever (including Typhoid and Paratyphoid) is a public health problem in the developing world, leading to approximately 14.3 million cases and 135.9 thousand deaths globally, with a significant number of cases in South Asia accounting for 71.8% of global cases in 2017.^1^ During a recent typhoid outbreak (2016 – 2018) in Pakistan, around two-thirds of reported cases were extensively drug-resistant (XDR) with case fatality rate of 7%.^2^,^3^

A common surgical complication of enteric fever caused by Salmonella enterica serotype Typhi or serotype Paratyphi is intestinal perforation which mostly affects terminal ileum. It has a better prognosis if occurs within first week.^4^ TIP less commonly affects the typhoid patients but is found to be the most common cause of peritonitis.^5^ It is not only increasing the morbidity and mortality of the disease but also creating worst impact on the economy of developing countries.^6^ However, early surgical management primarily within 24 hours, may cause a decrease in morbidity as well as mortality.^7^

In support with WHO, Pakistan has initiated various appreciable programs including initiation of immunization and control of emerging antibiotic resistance mainly XDR.^3^ In addition to these measures, adequate timely management is also essential, which is only possible by early diagnosis of the disease. Blood culture, although has 100% specificity, is neither rapid diagnostic nor widely available in resource limited setups. Moreover, its sensitivity decreases with previous antibiotic use, increased duration of disease and inadequate sample volume.^8^ Thus, an appropriate clinical diagnosis in initial course of disease should be considered a more reliable diagnostic tool.

Previous literature describes classical clinical picture of typhoid fever as, a low-grade fever with a continuous step ladder pattern that persists for three or more consecutive days, constipation, soup water diarrhea, and presentation of peritonitis due to TIP during third week of illness or in late second week.^4^,^9^

Recent findings indicate noticeable variations in the clinical presentation of the disease.^10^ In most of the studies conducted in Pakistan the atypical presentation is primarily either due to development of rare complications like hepatitis, cholecystitis, gastrointestinal bleeding, encephalitis, or some co-infections.^11^ However, for last decade paucity of studies are there addressing the change in clinical symptoms among the patients having typhoid related ileal perforation. Present study, specifically aimed at highlighting the frequency of those typhoid cases of TIP whose clinical history before evident peritonitis, was atypical and different from those described in literature.

## METHODOLOGY

A cross sectional study was conducted among patients admitted in surgical department JPMC, started from 20^th^ January 2021 to 20^th^ January 2024. Sample size of 98 was calculated using OpenEpi calculator putting estimated frequency of 28.7%, confidence interval of 95% and margin of error of 9%. Sample collection was done with non-probability consecutive sampling technique.

### Ethical approval:

It was taken from institutional IRB Ref. No: F.2-81/2024-GENL/40/JPMC; dated July 29, 2024.

### Inclusion criteria:

- Patients with age between 13 and 75 years, having clinical history and operative findings consistent with TIP, were included.

### Exclusion criteria:

- Patients who refused to consent, had previously undergone surgery or intervention and developed peritonitis as its complication, those presented with ileal perforation not attributable to typhoid (e.g. secondary to malignancy, tuberculosis etc.), pregnant females, prolonged steroid users, and patients with uncontrolled comorbidities, were excluded.

### Data collection:

Data was obtained from ninety-eight eligible cases of TIP. The diagnosis was confirmed by operating surgeon based on per-operative findings and supported by clinical history, examination and investigations. Each patient was approached by either the attending doctor or the study investigator. After obtaining informed verbal consent data was collected on self-designed proforma **(Annexure-I)** by the study investigator. Patient information was obtained through a detailed history and review of available investigations. Pre-operative laboratory investigations, including complete blood count, renal and liver function test, and coagulation profile, were advised to support the diagnosis and to optimize patient for planned surgery. The outcome was calculated as per operational definition.

**Figure:**
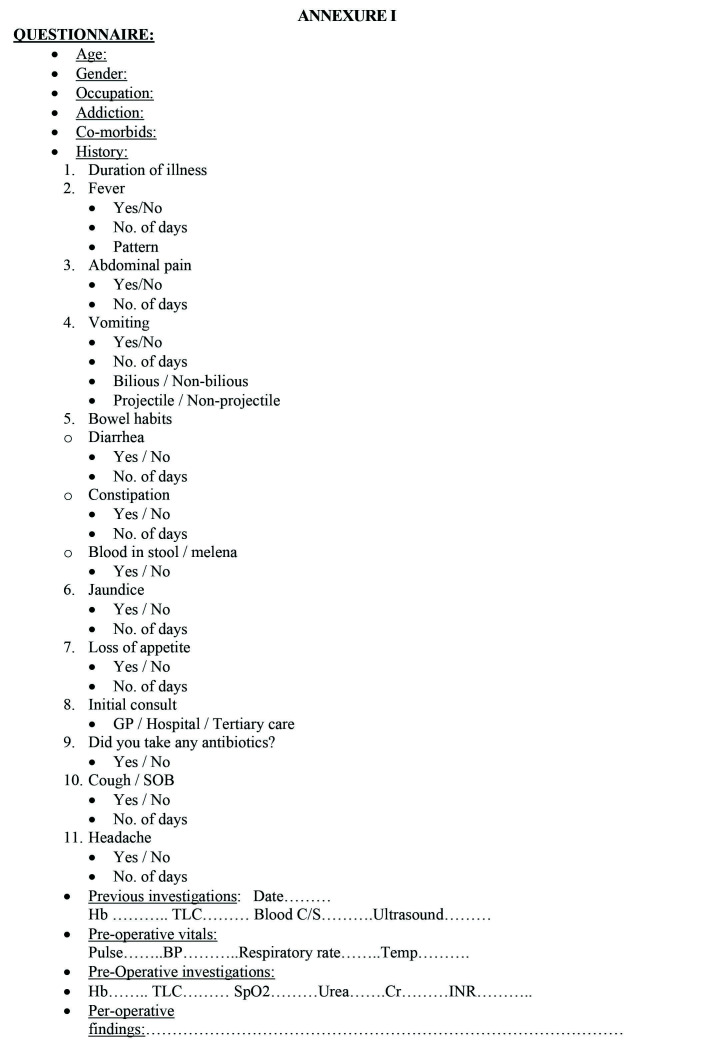


### Data analysis:

Data entry and analysis was done with the software SPSS version 25. Mean with standard deviation, median (IQR) and mode was calculated for age and duration of a symptom. Frequency with percentages was calculated for categorical data including gender, tobacco addiction, co-morbidities, presence, character and pattern of a symptom, initial consult and antibiotic use before presentation. Stratification was done to control the effect modifiers such as age, gender etc., to see their effect on outcome. Post stratification Chi-Square/ Fisher’s exact test was used. P-value of ≤ 0.05 was considered as significant.

### Operational Definitions:

Typhoid Intestinal Perforation (TIP) was defined per-operatively as, single or multiple perforated ulcers with friable margins (or ulcers with impending perforation or necrotic patches) arranged in parallel on anti-mesenteric border of the terminal ileum, associated with purulent or feculent contamination of peritoneal cavity. Atypical Clinical Presentation of Enteric Fever was defined as any deviation from literature explained typical clinical features of enteric fever, including duration of illness for two to three weeks, fever with continuous step ladder pattern and abnormal bowel habits, before evident peritonitis. All the operational definitions were evidenced by scientific literatures.^4^,^8^,^9^

## RESULTS

**Fig.1:**
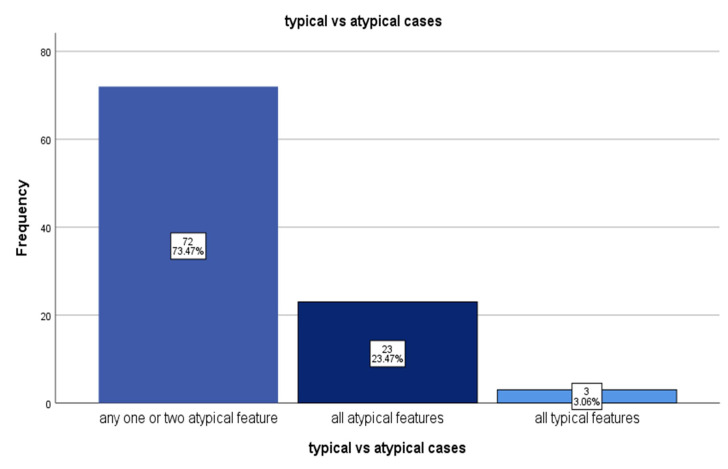
Typical vs. Atypical Typhoid Cases

The study shows 82.7% of the cases were male, 41.8% were addicted to tobacco, 10.2 % had co-morbidities and 45.9% had history of prior antibiotic consumption. Most common affected age group was 13 to 20 y, with median of 23 (18, 35) and mean ± SD of 27.29 (±12.46) years. Out of 98 number of enrolled cases with typhoid perforation, 95 number of cases (96.93%) had atypical clinical presentation, considering any one of the atypical features as an atypical case. Among them 23.49% (23) had all three atypical features and 73.47% (72) cases had one or two atypical features, as mentioned in Fig.1.

Atypical duration of illness i.e. ≤14 days or >21 days was found in 75.5% (74) patients. Majority of cases i.e. 38.8% (38) were reported during week-one, followed by week-two (22.45%), week-three (24.49%) and beyond week-three (14.28%). Atypical fever pattern or no fever was reported in 76.53% (70) of the cases. Among which 66.3%, 3.1% and 2% had intermittent, remittent and relapsing pattern of fever, respectively, and 5.1% patients were afebrile. No change in bowel habits was found in 41.84% (41) patients. Constipation and diarrhea were reported in 77.19% (44) and 22.81% (13) of the cases, respectively.

Considering p-value of ≤0.05 statistically significant, no significant effect of any of the effect modifiers including age, gender, addiction, co-morbidities, initial consultation and prior antibiotic use was found on any of the outcomes including frequency of atypical duration of illness, atypical bowel habits and atypical fever pattern or no fever. The most common symptom (97.96%) was abdominal pain. Patients also gave history of loss of appetite, vomiting, headache, blood in stool, jaundice and respiratory tract related symptoms with a frequency of 41.8% (41), 58.16% (57), 20.4% (20), 5.1% (5), 2% (2) and 8.16% (8), respectively.

## DISCUSSION

Recently enteric fever has been presented with variable clinical picture. Present study shows highest number of TIP cases in first week of illness i.e. 38.8% (38) that were around twice to those presented during the second or third week. Least number of cases i.e. 14 (14.28%) were reported after third week of illness. Whereas frequency was almost same in second week as well as in third week. Khanam, F. et al. also has recently reported a case of TIP having duration of only six days.^12^ On the contrary, Grema B.A. et al. in their study, reported lowest frequency of TIP during first week of illness and highest frequency during second week (59.5%).^13^

However, in accordance with multiple local and international recent studies^7^,^12^-^14^ our study also shows that majority of typhoid cases present with TIP within two weeks of illness, unlike previous literature.^4^,^9^ Early development of TIP, if managed early, has better prognostic value.^4^ On the other hand due to its unusual early presentation, this may lead to delay in referral to a tertiary care center for early surgical intervention, worsening the disease outcome.^7^

Fever was a predominant clinical feature of the present study, like all other local and international studies.^3^,^15^-^18^ It was found in 94.9% (93) of all enrolled cases. Only 23.47% (23) cases reported typical continuous step ladder pattern of fever, while 76.53% (70) did not follow the typical pattern. Intermittent fever pattern was the most common pattern noticed in our study, as reported by Nimonkar RA. et al.^18^ In the present study, 66.3 % (66) of the patients reported intermittent fever pattern, while only 3.1% (3) of cases had remittent and 2% (2) of cases had relapsing fever pattern. Contrary to this, Hemant Kumar et al. in a recent Indian study has reported that continuous fever pattern was found in 52.3 % cases, remittent pattern in 28.5% cases and intermittent pattern in only 19% of the studied cases.^19^

However, none of the cases with continuous pattern had step ladder rise in temperature.^19^ Patients who were afebrile have also been considered as atypical constituting 5.1% of the total cases, which is minimum in our study similar to most of the studies, except a recent local study in which slight increase in frequency of afebrile patients i.e.30% was seen.^18^ More than half i.e. 58.16% (57) of the cases had alteration in their bowel habit, a classical symptom of typhoid. The result was near to that found by researchers of SEAP study.^3^ The constipation was found 3.4 times more than diarrhea in our study. This is in accordance with most of the studies conducted among cases with TIP.^13^,^17^ and in contrary to a recent study reporting diarrhea as a predominant bowel habit in TIP cases.^7^

Abdominal pain was the most common presenting clinical feature of our study, like other studies done on TIP cases.^3^,^13^,^20^ Median (IQR) duration of pain was 3 (2, 7) days, more than half (56%) of the patients had duration between 1 and 3 days, and total twenty number of patients had duration of only one day. The short duration of pain is consistent with peritonitis in our study, as also evidenced by Yousafzai MT et al. finding pain more commonly in cases with TIP than those without TIP.^21^

### Strength of the study:

Reviewing the past decade reveals a lack of local research on changes in the clinical pattern and duration among TIP patients. This study, conducted at JPMC, one of Pakistan’s largest tertiary care hospitals, aimed to guide clinical diagnosis of typhoid. The study contributes to existing literature by highlighting variations in the disease’s clinical presentation with most cases showing early ileal perforation and intermittent fever, contrasting with standard descriptions. These results align with other local studies reporting atypical presentations. Hence, the clinical pattern of typhoid should be re-evaluated globally, especially in endemic regions, to update standard medical references. Early diagnosis and prompt surgical referral can significantly reduce morbidity and mortality of the disease.

### Limitations:

As the duration of illness was measured in days and not in weeks(s) this may cause some estimation error. However, the amount of estimation error doesn’t affect the results due to adequate sample size. Secondly, the diagnosis of typhoid perforation was made based on the patients’ history, immediately available test results and confirmation of typhoid on the basis of per-operative findings. The lack of a bacterial culture report to confirm typhoid-related ileal perforation might have resulted in over-estimation of cases in this study. However, as per WHO, blood cultures are less reliable in such cases due to delayed presentation and prior antibiotic use. In surgical emergencies like generalized peritonitis, laboratory tests aid diagnosis but should not delay surgery, where operative findings and clinical history remain central to diagnosis. Close collaboration with surgeons minimized diagnostic bias, and patients diagnosed with perforations due to tuberculosis, Crohn’s disease, or malignancy were excluded. Lastly, recall bias and poor fever documentation may have affected accuracy, but these were minimized through careful history taking and review of available medical records.

## CONCLUSION

The clinical spectrum of typhoid is vast, making its clinical diagnosis and management difficult. Some cases of typhoid perforation had shortest duration of illness of even one day, establishing TIP an inevitable complication. However early diagnosis and early referral for surgical management can definitely decrease the morbidity and mortality in these cases. Good hygienic practice, immunization and adequate treatment can definitely lead to decrease burden of typhoid and its related complications.

### Authors Contribution:

**TKR:** Data collection, statistical analysis & interpretation and manuscript writing and final approve of the manuscript.

**AY and GR:** Conceived, drafted, designed, reviewed and approved the manuscript.

**SMAJ:** Reviewed, did statistical analysis, manuscript writing and final approval of manuscript.

All authors are responsible and accountable for the accuracy or integrity of the work.
